# Aortic Arch Floating Thrombus Complicated by Distal Embolization in a Patient with Malignancy

**DOI:** 10.1155/2018/2040925

**Published:** 2018-10-02

**Authors:** Konstantinos Tigkiropoulos, Dimitrios Karamanos, Marianthi Tympanidou, Nikolaos Saratzis, Ioannis Lazaridis

**Affiliations:** Vascular Unit, 1st University Surgical Department, Aristotle University, Papageorgiou General Hospital, Thessaloniki, Greece

## Abstract

Free floating thrombus of aortic arch in a minimally atherosclerotic or nonaneurysmal aorta is a rare disease entity which carries a potential risk of distal embolization with catastrophic consequences. We present the case of a 52-years-old patient with ovarian cancer and aortic arch floating thrombus who initially managed with low molecular weight heparin and eventually undergone surgical thrombectomy of left external iliac and common femoral artery due to acute ischemia of left lower leg.

## 1. Case Report

**Figure 1:**
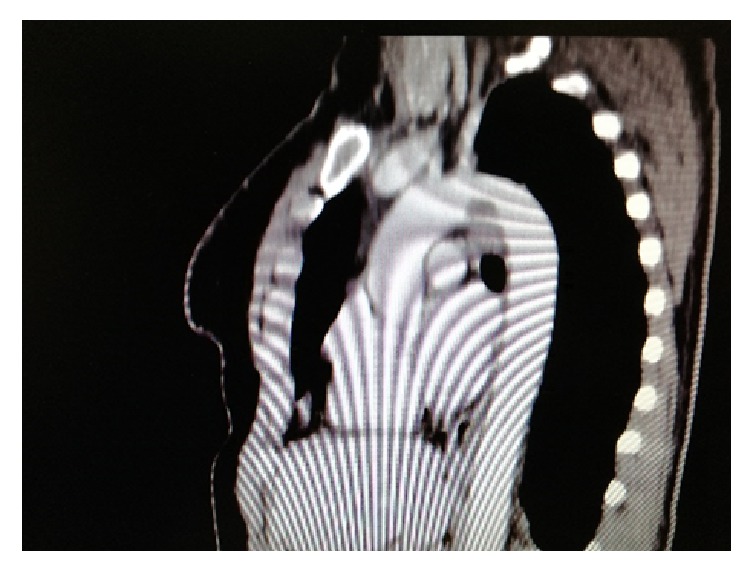
Coronal and axial view of aortic arch depicted the presence of thrombus in a calcified lesion.

**Figure 2:**
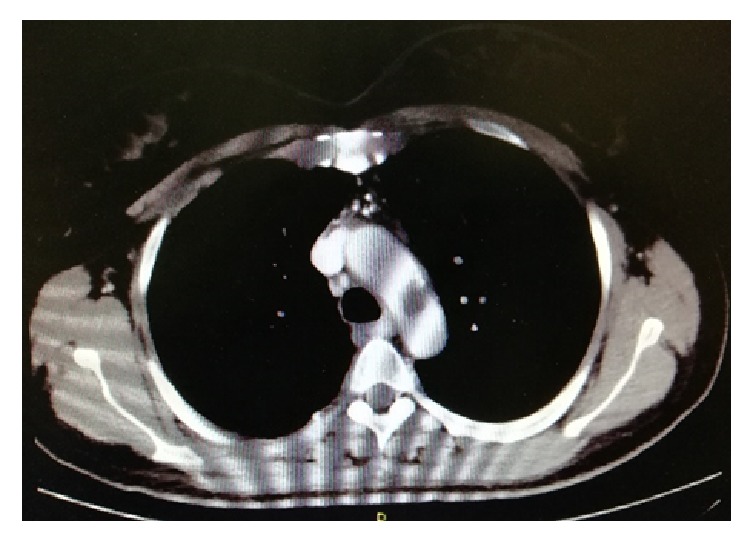
Coronal and axial view of aortic arch depicted the presence of thrombus in a calcified lesion.

**Figure 3:**
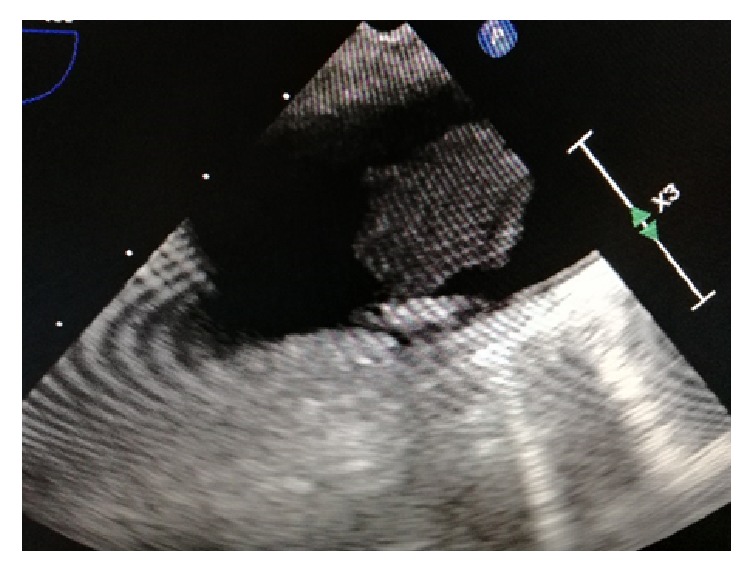
|Transesophageal echocardiography showed a 23 mm mobile thrombus in the lesser curvature of aortic arch.

**Figure 4:**
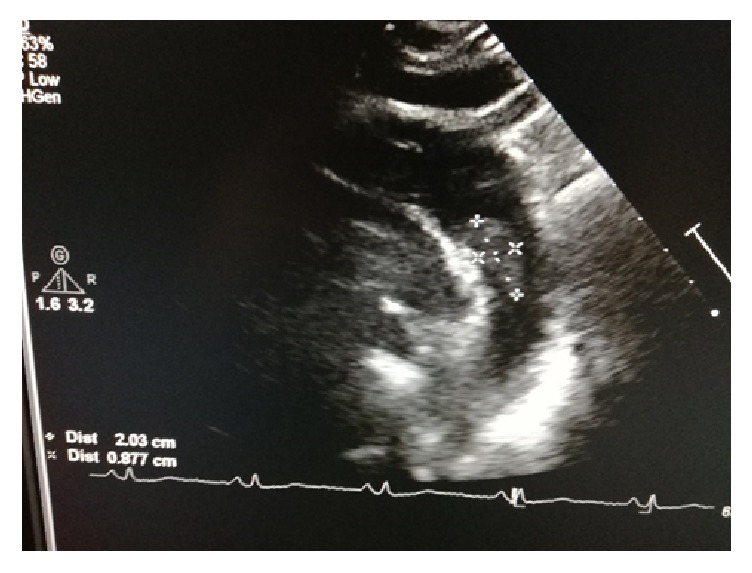
|Transesophageal echocardiography showed a 23 mm mobile thrombus in the lesser curvature of aortic arch.

A 52-years-old woman was hospitalized in the gynecological department due to recently diagnosed left ovarian cancer. Her medical history was unremarkable except from cigarrete smoking. Hematocrit was 38,1% and biochemical values of renal and hepatic function were within normal values. Tumor markers CA 15-3 was 39,5 U/ml (Normal Value (NV) <31,30), CA 19-9 was 104,93 U/ml (NV<37), and CA125 was 875,20 (NV<35 U/ml). A contrast-enhanced computed tomography (CT) of thorax was performed to exclude distal metastasis. It revealed a nonadherent thrombus of 20 mm long in the distal part of aortic arch (Figures 1 and 2). Immediate consult of vascular unit was scheduled and she was then referred to our department for further evaluation and treatment. Thrombophilia screen including Protein C, Protein S, Antithrombin III, APC Resistance-V, Factor VIII, homocysteine, and Anticardiolipin antibodies was performed which was negative. A transesophageal echocardiography was scheduled which showed a pedunculated free floating thrombus (FFT) 23 mm long of aortic arch (Figures 3 and 4, Video 1). The patient was commenced low molecular weight heparin therapy (LMWH) (enoxaparin, 8.000 units twice daily). Endovascular treatment was excluded since there was a high risk of distal embolization with manipulation of guidewires in the aortic arch and deployment of endograft. Open repair under extracorporeal cardiopulmonary bypass and hypothermic circulatory arrest from cardiothoracic surgeons was the second option but the patient unexpectedly denied fearing perioperative complications.

**Figure 5:**
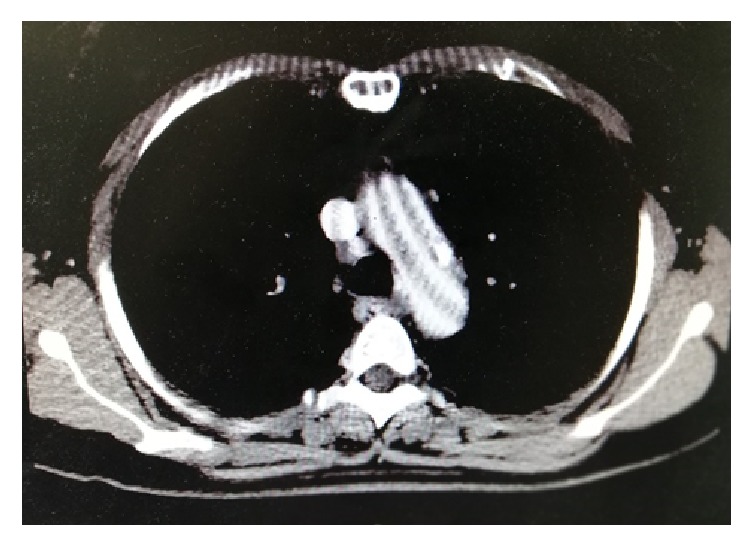
CT angiography revealed dislodgement of aortic thrombus and occlusion of the left common femoral artery (Absence of contrast agent in the arterial phase).

**Figure 6:**
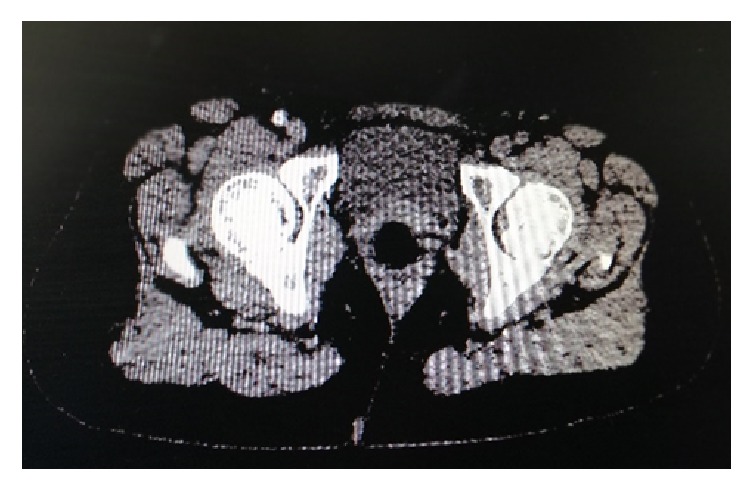
CT angiography revealed dislodgement of aortic thrombus and occlusion of the left common femoral artery (Absence of contrast agent in the arterial phase).

At the 8th day of her hospitalization patient experienced a sudden onset of pain in her left leg with incoming paresthesia and motion weakness. A provisional diagnosis of acute left leg ischemia was established and CT angiography of thoracic and abdominal aorta was performed which revealed complete dislodgement of thrombus from aortic arch which embolized as whole “thrombus” and thrombosis of left external iliac and common femoral artery (Figures 5 and 6). The patient was emergently transferred to the operation theatre where thromboembolectomy with Fogarty catheter was performed under local anesthesia. A histopathologic examination of the mass revealed the presence of exudative inflammatory cells (polymorphonuclear and lymphocytes) and fresh thrombus. No malignant cells were detected. The patient had an uneventful postoperative recovery and was discharged under enoxaparin, 8.000 units twice daily for at least one month in agreement with our Hemostasis specialist. During follow-up the patient remained asymptomatic and neoadjuvant chemotherapy was begun and operated for ovarian cancer 5 months later, under LMWH (Enoxaparin) 8.000 once a day.

## 2. Discussion

Mural thrombi within aortic aneurysms and ulcerated atherosclerotic plaques are the most frequent nonheart causes of embolization [1, 2]. However aortic thrombi in minimal/nonatherosclerotic and nonaneurysmal aorta is a rare disease entity which is frequently seen in younger patients without severe atherosclerosis [3–5]. That is why it must be separated from the typical atherosclerotic process that takes place in older patients. In these conditions hypercoagulable disorders including Protein C and S deficiency, Hyperhomocysteinemia, elevated levels of Factor VIII, Antithrombin III deficiency, polycythemia vera and Systemic Lupus Erythromatosus in addition to malignancy, trauma, and instrumentation are responsible for development of aortic floating thrombus [6–10]. Clinical presentations range from asymptomatic disease to symptoms related to devastating complications of cerebral, peripheral, or visceral embolization [3]. Embolization rate of floating and nonfloating thrombus has beenreported in 75% and 12% of patients, respectively [11]. Most cases of aortic thrombus are diagnosed after embolic events; however some are diagnosed incidentally during routine examinations. Pathophysiologic mechanisms which contribute to thrombus formation include turbulent blood flow, transient hypercoagulable state, and atherosclerotic process [12].

Imaging modality of choice for diagnosis of free floating thrombus includes CT aortography followed by transesophageal echocardiography (TEE). CTA permits visualization of thoracic and abdominal aorta, supra aortic branches, the presence of aortic wall calcifications, and distal embolization sites. Heart, ascending aorta, and aortic arch thrombus are well visualized by transesophageal echocardiography. It permits distinction of floating from adherent thrombus as well as atherosclerotic processes in the proximal part of the aorta [13].

The treatment of FFT in the aorta is still controversial and there is no consensus between authors regarding therapy recommendations [14, 15]. Anticoagulation, open surgical thrombectomy, and in certain cases endovascular therapy are the treatment options [16, 17]. American Heart Association (AHA) guidelines regarding prevention of stroke in patients with stroke and transient ischemic attack recommend that patients with adherent or mobile aortic arch thrombus should be treated medically to minimize cerebral embolic events rather than undergone an aortic arch endarterectomy or a cardiac procedure due to increased rate of intraoperative stroke [18]. Anticoagulation is suggested as a primary modality of therapy by many authors who reported complete resolution of thrombus without aortic arch surgical approach [16, 17]. Circulatory arrest and extracorporeal circulation are required for surgical thrombectomy in the aortic arch where a perioperative risk of devastating events, especially cerebral embolization, seems to be unexpectedly high in some reports [19].

On the contrary a more aggressive approach with open aortic surgical repair under extracorporeal circulation and hypothermic arrest has been proposed as a definite treatment of aortic arch thrombus. A meta-analysis of Fayad ZY et al. [4] showed that 200 patients (112 patients conservative treatment, 88 open surgical thrombectomy) with aortic mural thrombus in a normal and minimal atherosclerotic aorta surgical management had better outcome in recurrence/persistence of aortic thrombus and complication rates like limb loss than anticoagulation therapy alone. Additionally 25% of patients treated conservatively required secondary aorticsurgery to treat recurrence of peripheral arterial embolization. Weiss et al. [20] reported the outcome of 10 patients with aortic mobile thrombi in their referral vascular center. Open aortic arch thrombectomy under cardiopulmonary bypass and hypothermic circulatory arrest was performed in 6 patients with uneventful postoperative course with no recurrence or embolic complication.

Endovascular aortic surgery has become the treatment of choice in many aortic pathologies including aneurysm, dissection, penetrating atherosclerotic ulcer, traumatic dissection, and intramural hematoma. However few reports have been documented regarding treatment of mobile aortic thrombi with endovascular stent graft [21–26]. Despite the fact that the outcome was favourable in all cases, the risk of distal embolization during guidewire manipulation and stent graft repair could be an “obstacle” for endovascular treatment.

In our case patient has a recently diagnosed left ovarian cancer and history of cigarette smoking. Hypercoagulability due to malignancy in association with early atherosclerotic lesions in the aortic arch which act as a nidus for thrombus formation could be the pathogenesis of aortic arch mobile thrombi. Patients with cancer have a higher risk of thrombus formation in arterial and venous system. Unfractionated Heparin (UFH) and LMWH are the main types of antithrombotic treatment. UFH action requests close monitoring of activated partial thromboplastin time (aPTT) to achieve an adequate therapeutic effect, every 6 hours initially and then daily to maintain its efficacy, in contrary to patients who are receiving LMWHs where laboratory monitoring is not typically required.

Despite immediate treatment with LMWH in therapeutic dose there was no resolution of thrombus and patient's experienced acute ischemia of left lower leg with was successfully managed with Fogarty thrombectomy without devastating complications.

## 3. Conclusion

Aortic arch thrombus in a normal or minimally atherosclerotic aorta is a rare cause of distal embolization with cerebral, visceral, and peripheral emboli increasing significantly morbidity and mortality. Diagnostic evaluation with CT angiography followed by transesophageal echocardiography is mandatory. Despite the fact that there is no consensus regarding optimal treatment, open surgical aortic repair, or anticoagulation therapy, it seems that conservative treatment should be used only in high risk and older patients who are unfit for surgery.

## Supplementary Materials

Supplementary Materials Video 1: Transesophageal echocardiography revealed the presence of free floating thrombus in a calcified lesion at the distal aortic arch.

## References

[1] Lau L. S., Blanchard D. G., Hye R. J. (1997). Diagnosis and management of patients with peripheral macroemboli from thoracic aortic pathology. *Annals of Vascular Surgery*.

[2] Reber P. U., Patel A. G., Stauffer E., Muller M. F., Do D. D., Kniemeyer H. W. (1999). Mural aortic thrombi: An important cause of peripheral embolization. *Journal of Vascular Surgery*.

[3] Laperche T., Laurian C., Roudaut R., Steg P. G. (1997). Mobile thromboses of the aortic arch without aortic debris: A transesophageal echocardiographic finding associated with unexplained arterial embolism. *Circulation*.

[4] Fayad Z. Y., Semaan E., Fahoum B., Briggs M., Tortolani A., D'Ayala M. (2013). Aortic mural thrombus in the normal or minimally atherosclerotic aorta. *Annals of Vascular Surgery*.

[5] Tsilimparis N., Hanack U., Pisimisis G., Yousefi S., Wintzer C., Rückert R. I. (2011). Thrombus in the non-aneurysmal, non-atherosclerotic descending thoracic aorta - An unusual source of arterial embolism. *European Journal of Vascular and Endovascular Surgery*.

[6] Berneder S., Van Ingen G., Eigel P. (2006). Arch thrombus formation in an apparently normal aorta as a source for recurrent peripheral embolization. *The Thoracic and Cardiovascular Surgeon*.

[7] Onwuanyi A., Sachdeva R., Hamiram K., Islam M., Parris R. (2001). Multiple aortic thrombi associated with protein C and S deficiency. *Mayo Clinic Proceedings*.

[8] Shapiro M. E., Rodvien R., Bauer K. A., Salzman E. W. (1981). Acute Aortic Thrombosis in Antithrombin III Deficiency. *Journal of the American Medical Association*.

[9] Hanson J. A., Lloyd M. E., Hughes G. R. V. (1994). Aortic root thrombus causing stroke in a patient with systemic lupus erythematosus. *Scandinavian Journal of Rheumatology*.

[10] Geha A. S., El-Zein C., Massad M. G. (2004). Surgery for aortic arch thrombosis. *The Thoracic and Cardiovascular Surgeon*.

[11] Karalis D. G., Chandrasekaran K., Victor M. F., Ross J. J., Mintz G. S. (1991). Recognition and embolic potential of intraaortic atherosclerotic debris. *Journal of the American College of Cardiology*.

[12] Kalangos A., Baldovinos A., Vuille C., Montessuit M., Faidutti B. (1997). Floating thrombus in the ascending aorta: A rare cause of peripheral emboli. *Journal of Vascular Surgery*.

[13] Soleimani A., Marzban M., Sahebjam M., Shirani S., Sotoudeh-Anvari M., Abbasi A. (2008). Floating thrombus in the aortic arch as an origin of simultaneous peripheral emboli. *Journal of Cardiac Surgery*.

[14] Erbel R., Aboyans V., Boileau C. (2014). 2014 ESC guidelines on the diagnosis and treatment of aortic diseases: document covering acute and chronic aortic diseases of the thoracic and abdominal aorta of the adult. The Task Force for the Diagnosis and Treatment of Aortic Diseases of the European Society of Cardiology (ESC). *European Heart Journal*.

[15] Sanon S., Phung M. K., Lentz R. (2009). Floating, Non-Occlusive, Mobile Aortic Thrombus and Splenic Infarction Associated With Protein C Deficiency. *Journal of the American Society of Echocardiography*.

[16] Stöllberger C., Kopsa W., Finsterer J. (2001). Resolution of an aortic thrombus under anticoagulant therapy. *European Journal of Cardio-Thoracic Surgery*.

[17] Bowdish M. E., Weaver F. A., Liebman H. A., Rowe V. L., Hood D. B. (2002). Anticoagulation is an effective treatment for aortic mural thrombi. *Journal of Vascular Surgery*.

[18] Kernan W. N., Ovbiagele B., Black H. R. (2014). Guidelines for the prevention of stroke in patients withstroke and transient ischemic attack: a guideline for healthcare professionalsfrom the American Heart Association/American Stroke Association. *Stroke*.

[19] Stern A., Tunick P. A., Culliford A. T. (1999). Protruding aortic arch atheromas: risk of stroke during heart surgery with and without aortic arch endarterectomy. *American Heart Journal*.

[20] Weiss S., Bühlmann R., von Allmen R. S. (2016). Management of floating thrombus in the aortic arch. *The Journal of Thoracic and Cardiovascular Surgery*.

[21] Reineke D. C., Grapow M. T. R., Schumann M., Seeberger M. D., Carrel T. P. (2009). Massive intraoperative thrombus of the aortic arch and proximal descending aorta: Case reports. *Journal of Cardiac Surgery*.

[22] Altenbernd J., Schürmann K., Walterbusch G. (2008). Stent graft therapy in mobile thrombus of the thoracic aorta. *RöFo*.

[23] Fueglistaler P., Wolff T., Guerke L., Stierli P., Eugster T. (2005). Endovascular stent graft for symptomatic mobile thrombus of the thoracic aorta. *Journal of Vascular Surgery*.

[24] Luebke T., Aleksic M., Brunkwall J. (2008). Endovascular Therapy of a Symptomatic Mobile Thrombus of the Thoracic Aorta. *European Journal of Vascular and Endovascular Surgery*.

[25] Piffaretti G., Tozzi M., Caronno R., Castelli P. (2007). Endovascular treatment for mobile thrombus of the thoracic aorta. *European Journal of Cardio-Thoracic Surgery*.

[26] Rancic Z., Pfammatter T., Lachat M., Frauenfelder T., Veith F. J., Mayer D. (2009). Floating aortic arch thrombus involving the supraaortic trunks: Successful treatment with supra-aortic debranching and antegrade endograft implantation. *Journal of Vascular Surgery*.

